# Executive function training in chronic traumatic brain injury patients: study protocol

**DOI:** 10.1186/s13063-019-3526-x

**Published:** 2019-07-15

**Authors:** Daniel C. Krawczyk, Kihwan Han, David Martinez, Jelena Rakic, Matthew J. Kmiecik, Zhengsi Chang, Linda Nguyen, Michael Lundie, Richard C. Cole, Marielle Nagele, Nyaz Didehbani

**Affiliations:** 1 Center for BrainHealth, The University of Texas at Dallas, 2200 Mockingbird Lane, Dallas, TX 75235 USA; 2 Department of Psychiatry, University of Texas Southwestern Medical Center at Dallas, NE 210, 5323 Harry Hines Blvd., Dallas, TX 75390 USA

**Keywords:** Cognitive rehabilitation, Traumatic brain injury, Executive functions, Daily life functions, Memory, Attention, Planning

## Abstract

**Background:**

Some individuals who sustain traumatic brain injuries (TBIs) continue to experience significant cognitive impairments chronically (months to years post injury). Many tests of executive function are insensitive to these executive function impairments, as such impairments may only appear during complex daily life conditions. Daily life often requires us to divide our attention and focus on abstract goals. In the current study, we compare the effects of two 1-month electronic cognitive rehabilitation programs for individuals with chronic TBI. The active program (Expedition: Strategic Advantage) focuses on improving goal-directed executive functions including working memory, planning, long-term memory, and inhibitory control by challenging participants to accomplish life-like cognitive simulations. The challenge level of the simulations increases in accordance with participant achievement. The control intervention (Expedition: Informational Advantage) is identical to the active program; however, the cognitive demand level is capped, preventing participants from advancing beyond a set level. We will evaluate these interventions with a military veteran TBI population.

**Methods/design:**

One hundred individuals will be enrolled in this double-blinded clinical trial (all participants and testers are blinded to condition). Each individual will be randomly assigned to one of two interventions. The primary anticipated outcomes are improvement of daily life cognitive function skills and daily life functions. These are measured by a daily life performance task, which tests cognitive skills, and a survey that evaluates daily life functions. Secondary outcomes are also predicted to include improvements in working memory, attention, planning, and inhibitory control as measured by a neuropsychological test battery. Lastly, neuroimaging measures will be used to evaluate changes in brain networks supporting cognition pre and post intervention.

**Discussion:**

We will test whether electronically delivered cognitive rehabilitation aimed at improving daily life functional skills will provide cognitive and daily life functional improvements for individuals in the chronic phase of TBI recovery (greater than 3 months post injury). We aim to better understand the cognitive processes involved in recovery and the characteristics of individuals most likely to benefit. This study will also address the potential to observe generalizability or to transfer from a software-based cognitive training tool toward daily life improvement.

**Trial registration:**

ClinicalTrials.gov, NCT03704116. Retrospectively registered on 12 Oct 2018.

## Background

Traumatic brain injury (TBI) is a major cause of death and disability [1]. TBI incidents commonly occur due to vehicle accidents, falls, and assaults [2]. TBI is also considered a signature injury of the wars in Iraq and Afghanistan [3], with as many as 25% of military personnel experiencing head or neck injuries and as many as 18% experiencing mild TBIs [4]. The effects of TBI include both physical and psychological symptoms. Physical challenges include frequent headaches, pain, sleep disturbances, and altered mood states that can negatively affect tasks of daily life [5]. Common cognitive symptoms following a TBI include difficulties with focused attention, working memory, long-term memory, and inhibitory control [6]. These cognitive symptoms can have additional impact on real-world tasks including job performance, social engagement, managing finances, shopping, and scheduling.

TBI survivors, including those with mild-to-moderate injuries, may continue to exhibit cognitive difficulties in functioning relative to optimal levels at work, home, or in the community even years post injury [4, 7]. A significant number of individuals do not fully recover cognitively after a brain injury. Lingering cognitive impairments can affect executive functions, which are dependent upon the frontal lobes and their associated white matter connections. Diffuse axonal injury (DAI) [8] is a term that describes white matter injuries experienced due to head trauma. These disruptions to white matter can further affect the connectivity of the brain, resulting in continued cognitive challenges [9].

The cognitive symptoms commonly experienced following TBIs are often considered deficits of executive function. Executive functions include working memory, planning, and cognitive control. We apply the terminology introduced by Burgess et al. [10] in order to clarify the TBI-related cognitive impairments targeted in this study. Burgess et al. referred to theoretical cognitive abilities as *constructs*. A construct can be operationalized into a task that allows it to be measured in terms of its *operations*. For example, an experimental measure involving maintaining a set of digits for a brief time is an operation-level description of the working memory construct. Constructs, such as working memory, influence daily life abilities at a *function* level. An example of a function-level description of the working memory construct is managing a set of items on a recipe list when preparing to cook a meal. In the current study, we focus on rehabilitation of cognition at the function level, while gathering measurements at the operations level, and targeting particular constructs affected in cognition.

TBI-related cognitive symptoms vary across individuals. Applying Burgess et al.’s [10] terms (constructs, operations, and functions), we aim our intervention at addressing the constructs affected in an individual at the level at which they are impaired (e.g. functional daily living, operational cognitive tasks, or both). This approach is sensitive to the varied nature of cognitive impairments that follow from TBIs. It is likely that training multiple cognitive functions that are directly relevant to functional impairments will provide maximal benefit to the most individuals.

We propose a study aimed at improving cognitive difficulties in individuals with chronic TBI (at least 3 months post injury) through training on cognitive tasks set within the context of carrying out daily life functions. The interventions will be delivered remotely through game-based simulations of different situations that are commonly encountered when traveling. The simulations allow the participants to accomplish cognitive tasks in travel environments that allow both modulation of task difficulty and collection of precise quantitative performance metrics. The remote deployment of the interventions will be accomplished using laptop computers, increasing accessibility for individuals who struggle with attending clinical appointments and for individuals for whom transportation, or work constraints, render in-office visits challenging. Improvements in daily life abilities may influence the family lives of the individuals we are able to reach, as better daily life goal management, task performance, and task scheduling can have a positive impact for spouses, caregivers, and families [11].

The overall goal of this trial is to examine how training multiple core cognitive functions through simulated daily life challenges may improve cognition for individuals coping with cognitive challenges caused by TBI. The active intervention, called Expedition: Strategic Advantage (ESA), is compared to a control intervention, called Expedition: Informational Advantage (EIA). EIA is identical in content to ESA but capped at a prespecified achievement level set for each module, thereby testing the efficacy of individually sensitive increases in challenge level. To measure efficacy, we will use a series of assessment tools, including cognitive, neuroimaging, and functional assessment measures, and surveys to compare outcomes and performance for the active and control interventions.

### Aims

The primary goal is to improve the functional recovery of individuals who have experienced cognitive challenges related to a TBI at least 3 months before enrollment. This study will also enable us to determine the effects of trained and untrained cognitive skills, neurological changes, and measures of daily life functional recovery.

#### Aim 1

Examine the effects of ESA compared to EIA on daily life outcomes in a military veteran population with chronic TBI.

##### Hypotheses related to Aim 1:

A. Participants enrolled in ESA will show a greater increase from pretest to post-test on measures of daily life function (the Virtual Multiple Errands Test (VMET) [12] and the TBI Awareness Questionnaire [13]) relative to participants randomized to EIA.

#### Aim 2

Examine the effects of ESA compared to EIA on cognitive outcomes in a military veteran chronic TBI population.

##### Hypotheses related to Aim 2:

A. Participants enrolled in ESA will show greater improvements in cognitive measures (Automated Neuropsychological Assessment Metrics (ANAM) battery measures (Matching to Sample, Mathematical Processing, Code Substitution, Stroop, and Tower Puzzle) [14], the Trail Making Test [15, 16], and the Similar Situations Task [17]) compared to EIA participants.

#### Aim 3

Examine changes in magnetic resonance imaging (MRI) measures to characterize participants and evaluate neural plasticity related to ESA compared to EIA participants.

##### Hypotheses related to Aim 3:

A. Participants showing greatest daily life and cognitive improvement after ESA will show changes in white matter microstructure associated with healthy plasticity (as measured by diffusion tensor imaging (DTI)).
B. Participants showing greatest daily life and cognitive improvement after ESA will show greater within and between-network coherence (as measured by resting-state functional magnetic resonance imaging (rsfMRI)).
C. Participants showing greatest daily life and cognitive improvement after ESA will show greater attention-related enhancement and suppression effects (as measured by functional magnetic resonance imaging (fMRI)).

## Methods

### Design

This is a two-arm, randomized, double-blinded (with respect to scoring and data analysis), single-center, controlled clinical trial. All participants will provide written informed consent before participating in any study procedures. Participants will be randomly assigned to one of the two intervention conditions (ESA or EIA).

**Fig. 1:**
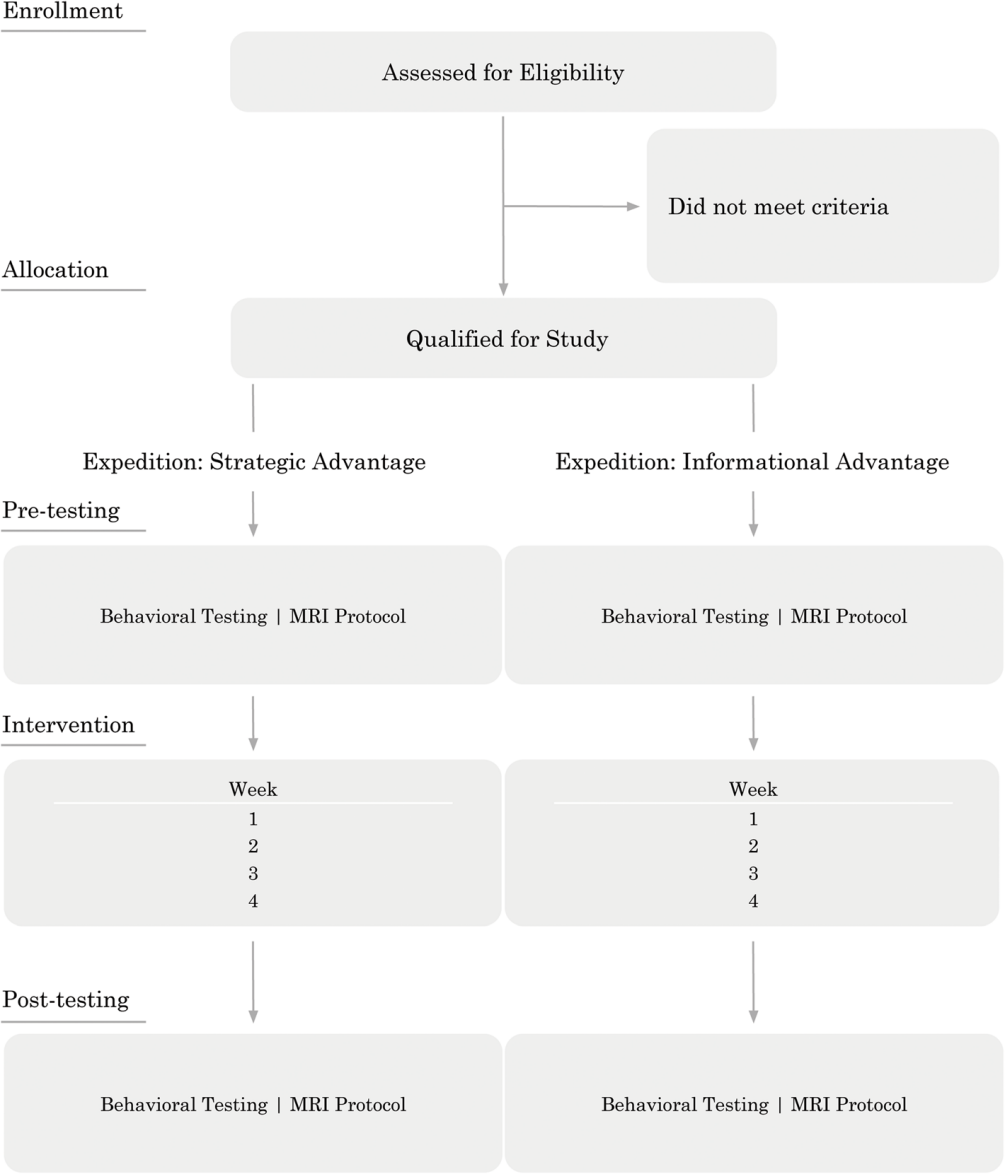
CONSORT diagram: flow chart listing each phase of the study experienced by the participants. The chart also contains summary information about the procedures followed by the experimenters in implementing the study. MR magnetic resonance imaging

**Fig. 2:**
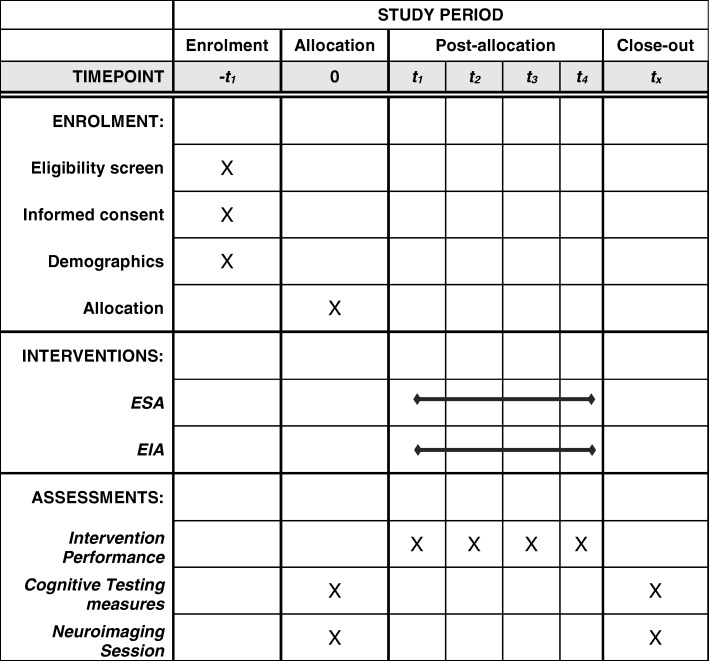
SPIRIT figure including a schedule of enrolment, interventions, and assessments. EIA Expedition: Informational Advantage, ESA Expedition: Strategic Advantage, SPIRIT Standard Protocol Items: Recommendations for Interventional Trials

The study has three phases: pretesting, intervention, and post-testing (see Figs. 1 and 2). In the pretesting phase, we collect demographic information including psychological assessments, neuropsychological assessments, cognitive assessments, MRI data, and daily life measures (see Table 1 for the measures list). The pretesting is conducted at two adjacent university sites located in Dallas, TX, USA. The intervention consists of two arms (ESA and EIA) and is administered via laptop computer (assisted by an experimenter when needed during eight check-in sessions) over the course of 4 weeks. The experimenter checks in with each participant at the beginning and mid-point of each week during the intervention. The experimenter check-in ensures that participants are putting in sufficient time on the intervention each week and enables the participant to ask for clarifications on their performance as needed. The remote laptop delivery enables these interventions to be mobile and to occur at any location. The post-testing phase largely repeats the testing procedures carried out at our Dallas, TX study site (psychological, neuropsychological, and cognitive assessments, MRI data, and daily life measures (see Table 1)). When possible, we employ alternate versions of tests to minimize practice effects in the post-testing session (the Trail Making Test is the only measure we use that does not enable multiple versions for test–retest purposes, although we anticipate that practice effects may occur for some measures).

**Table 1:** Tests and estimated duration

Test	Duration
A. Function/dysfunction tests (self-report measures)	Approximately 60 min
1. Measures	
1. Post-Traumatic Stress Disorder Checklist (PCL-S)	<5 min
2. OSU TBI Screen Form	10–15 min
3. Demographic/medical form/WARCAT	10–15 min
4. Awareness Questionnaire	<5 min
5. Glasgow Outcome Scale—Extended	<5 min
6. Beck Depressive Inventory	5–10 min
7. Beck Anxiety Inventory	5–10 min
8. AUDIT (alcohol use)	<5 min
9. ASSIST (drug use)	5–10 min
B. Estimate of current IQ	
1. Test of Premorbid Functioning	5–10 min
C. Working memory	
1. Matching to Sample task (ANAM)	
2. Mathematical Processing task (ANAM)	
D. Long-term memory/recall	
1. Code Substitution (ANAM) (processing speed and memory)	
E. Processing speed	
1. Trail Making Test—A (connect numbers only)	Few minutes
F. Attention	
1. Stroop (ANAM)	
G. Inhibition/switching	
1. Trail Making Test—B (connect number–letter–number, etc.)	Few minutes
H. Reasoning	
1. Similar Situations Task	Cut in half, 15 min
I. Planning	
1. Tower Puzzle task (ANAM)	
J. Intervention	
1. Virtual Multiple Errands Test	15–20 min

*WARCAT* Warrior Administered Retrospective Casualty Assessment Tool

### Participants

We will recruit 100 participants (age 18–55 years) with a history of mild-to-moderate TBI as indexed by the Ohio State University (OSU) TBI assessment [18]. Participants will be veterans of the US military. Participants will only include people who can speak and comprehend English, as not all of the standardized and experimental cognitive tests have been normed for non-English speakers. This study has been approved for research with human subjects by the Institutional Review Boards (IRBs) of the University of Texas Southwestern Medical Center at Dallas (IRB#8843) and The University of Texas at Dallas (IRB#11-43) and complies with the Declaration of Helsinki.

### Inclusion criteria

Selection criteria include male and female veterans who have sustained a TBI at least 3 months previously, can comprehend simple instructions, can perform the tests, and can take part in the interventions. Participants will be screened and enrolled at the Center for BrainHealth® of The University of Texas at Dallas. Selection criteria also include participants who can safely undergo MRI (although lack of this is not exclusionary), who can tolerate at least 2 h of testing sessions at a time, and who can participate in tasks involving motor abilities such as the use of at least one arm and hand. No racial/ethnic groups will be excluded, although the participants must be able to speak, read, and comprehend English sufficiently to complete testing sessions.

### Exclusion criteria

Exclusionary criteria include lack of proficiency in reading, comprehending, and speaking English, preexisting cerebral palsy, mental retardation, autism, schizophrenia, pervasive developmental disorder, major depression, psychosis, active behavioral disorder, or uncontrolled epilepsy. Many individuals who have sustained a TBI during military service also express post-traumatic stress disorder (PTSD) symptoms. We are sensitive to these and measure their presence, using the PTSD Checklist (PCL) [19], but endorsement of some symptom level is not exclusionary. Optimally, all participants will be capable of undergoing MRI, but due to the possibility of shrapnel or metal-related injuries within the sample population, lack of this is not exclusionary to participation (without MRI). Participants cannot be actively engaged in another cognitive intervention concurrently.

### Feasibility estimate

Both performance sites, The Center for BrainHealth® and the Brain Performance Institute, are centrally located in the Dallas–Fort Worth metropolitan area, allowing access to a large and diverse population including numerous individuals who have sustained TBIs either in or outside military service. Over the past 6 months, we have been able to recruit an average of four to five individuals per month who qualify for the study. This level of patient flow indicates that we should be able to meet our sample size within 2 years, meeting the goals for the project-funding period. We will make information about the study available via clinicaltrials.gov, veteran social media (Facebook and Instagram sites relevant to veteran populations), the Internet (http://www.project-expedition.org), and veteran events (university and college veteran center fairs/meetings).

### Screening

Participants are screened for inclusion/exclusion criteria during a structured interview, which includes use of the OSU TBI assessment, the Glasgow Outcome Scale—Extended (GOS-E) [20], and a set of relevant demographic, medical, PTSD, and TBI questionnaires. The presence of PTSD symptoms is screened for in the current study, but history of PTSD symptoms is not exclusionary (as noted earlier). In addition to the severity level indicated by the OSU TBI assessment, we seek confirmatory evidence based on Glasgow Coma Scale (GCS) [21] documentation of prior medical evaluations when available, although lack of a documented GCS is not exclusionary. Given the limited availability of GCS scores, particularly for mild TBI participants, we ultimately rely on GOS-E functional severity and OSU TBI assessment to define TBI severity.

### Primary outcome measures

The VMET and TBI Awareness Questionnaire are the primary outcome measures.

The VMET is an executive function measure implemented in a game-based simulation and is based on Shallice and Burgess’ Multiple Errands Test [22] that was carried out in real-world environments. It requires participants to plan and execute several task goals within a set of predefined rules. Success depends on implementing memory, inhibitory control, and planning needs in a novel context. Greater performance on this measure for the ESA group over the EIA group would represent “near transfer” of executive function improvements in a game-based simulated context [23]. VMET data are scored by tallying the total numbers of task failures, inefficiencies, strategies, rule-breaks, and interpretation failures [12]. A task failure occurs when a subtask is not completed satisfactorily. An inefficiency occurs when a more effective strategy could have been used in order to accomplish the task. Strategies can be used that facilitate carrying out the tasks, and the use of possible strategies is scored based on their effectiveness. Rule-breaks represent violation of a rules-set that are listed on the task instructions sheet. Interpretation failures are cases in which the requirements of a particular task are misunderstood.

The TBI Awareness Questionnaire [13] is a survey measure asking participants to evaluate their cognitive abilities at the current time compared to pre injury and serves as another “far transfer” real-world evaluation of current TBI impairment. We predict that the ESA will improve TBI symptom recovery compared to EIA as measured by this instrument.

### Secondary outcome measures

Secondary outcome measures are employed to characterize participants’ cognitive and emotional functioning and to address changes in cognition related to the interventions. The GOS-E is included (the initial GOS-E score is used to assess TBI symptom severity, the second administration is an indication of change from initial baseline). The OSU TBI assessment is used to assess TBI history and severity. The PCL—Specific (PCL-S) is used to screen for post-traumatic stress disorder (PTSD) symptoms. We assess depression and anxiety using the Beck Depression Inventory (BDI) [24] and the Beck Anxiety Inventory (BAI) [25]. The Alcohol Use Disorders Identification Test (AUDIT) [26] and the alcohol, smoking and substance involvement screening test (ASSIST) [27] are used to screen for substance use history. The Test of Premorbid Functioning (TOPF) [28] is given to assess pre-TBI intelligence. Cognitive executive function measures are gathered using the ANAM (Matching-to-sample Test, Mathematical Processing Test, Code Substitution Test, Stroop test, and Tower Puzzle) along with the Trail Making Tests (part A and B) and the Similar Situations Task.

### Interventions

In the current randomized control trial, we study the efficacy of a functional cognitive training software tool applied to individuals experiencing cognitive deficits due to chronic TBI. The program is delivered via four separate simulation modules over the course of 1 month. There are over 100 different possible specific playable instances within each module, enabling the intervention to remain novel for the participant throughout the month of training. All navigation occurs within game-based simulations using a mouse. All interactions are achieved by dragging, pointing, and clicking the mouse. This mouse interface is designed to be easy for participants to use and was adopted to avoid human–computer interaction challenges that some individuals may experience when using a virtual reality headset or specialized gaming controller. Each simulation enables increased challenge to be delivered based on increasing the complexity of the simulations being delivered to the participant. The simulations scale in difficulty according to participant achievement. The level of challenge will be increased over the course of repeated success, while the challenge level will be reduced if a participant repeatedly performs poorly on a given module over several playable instances.

Smartphone usage is nearly ubiquitous in the USA at the time of this study. In accordance with this trend, all modules include an electronic version of a smartphone displayed in the lower left corner of each simulation. The phone serves two potential functions. First, it can help participants with information, much like a real-world smartphone. Second, it can distract participants, again mimicking the capabilities of a real-world smartphone. Participants are encouraged to use the smartphone for helpful purposes, but to minimize its capability for distraction by selectively ignoring it when it provides no advantage to task completion.

#### Week 1

The first week focuses on the *construct* prospective working memory improvement. This is implemented through the *operation* of gathering a set of items that meet the task goals. These are applied to the *function* of simulated packing for a vacation or work-related trip. Participants navigate a small apartment (bedroom, closet, and bathroom) and are challenged to meet a set of goals for their trip by obtaining goal-relevant clothing and travel-related items from a series of closets and dressers. Items that may be selected for packing can be organized in a staging area and transferred to a set of virtual suitcases by clicking, dragging, and dropping the items.

**Fig. 3:**
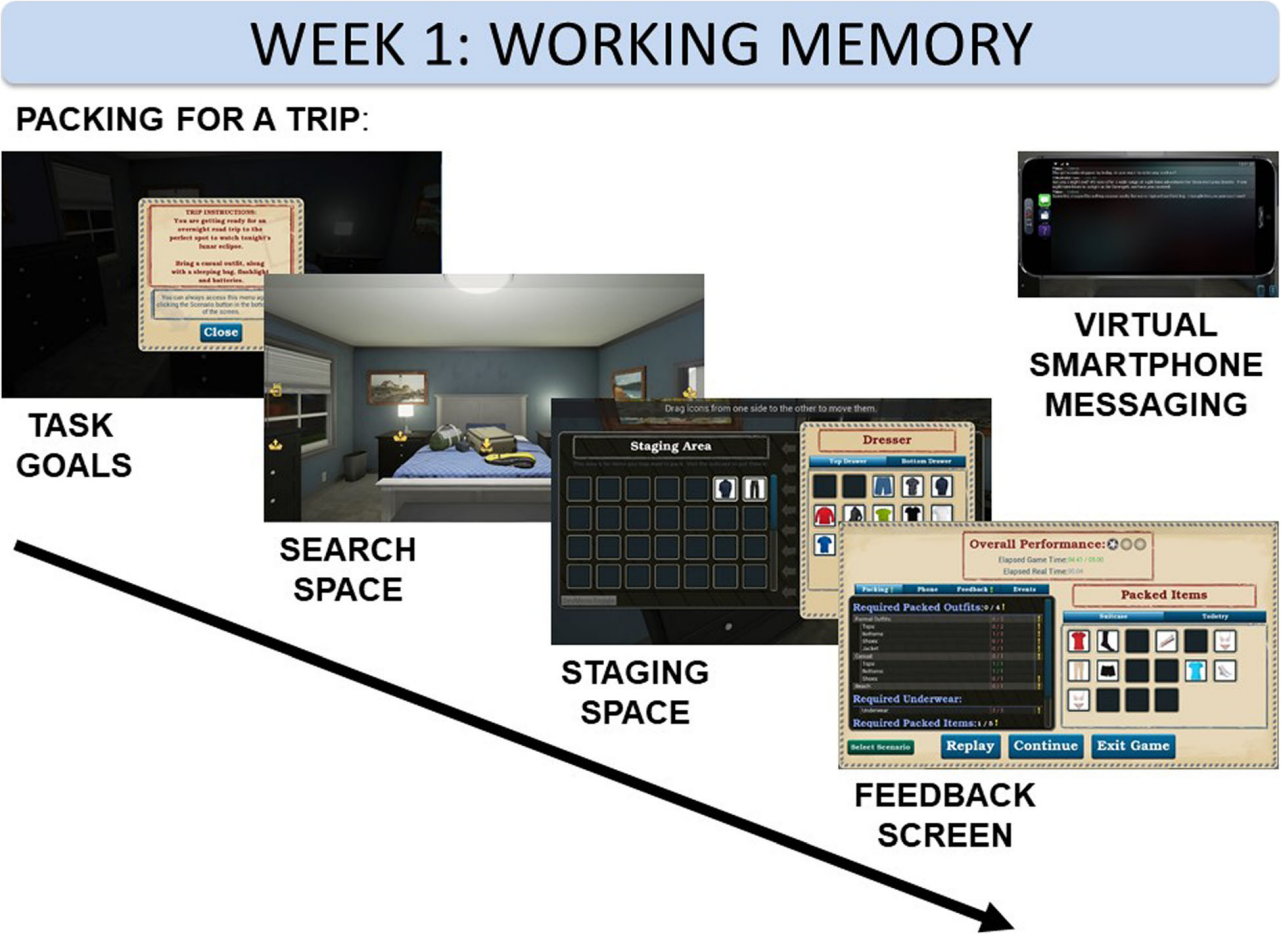
The working memory module begins with a series of task goals that specify the constraints of the current packing assignment. Next, participants navigate a virtual bedroom and can check cabinets and closets for items to be placed into a staging space. Lastly, participants complete their packing into virtual suitcases and receive a score for their performance. Throughout, participants receive potentially helpful and distracting text messages on a virtual phone

Working memory demand increases as participants succeed on the task (through accuracy and speed). If task demands are met multiple times (based on a proficiency algorithm), the difficulty scales up. This involves the addition of more items needed to successfully pack for progressively longer trips (the number of days that the trip specifies in an instructions screen), adding to the overall item load, and greater complexity (e.g., business presentation and side trip to an exotic location requiring specific items) (see Fig. 3).

The smartphone in week 1 serves the helpful function of providing texts to remind participants of certain task requirements to focus on as they pack. The phone also distracts from successful performance by buzzing irrelevant text messages to the participant throughout the simulation.

#### Week 2

**Fig. 4:**
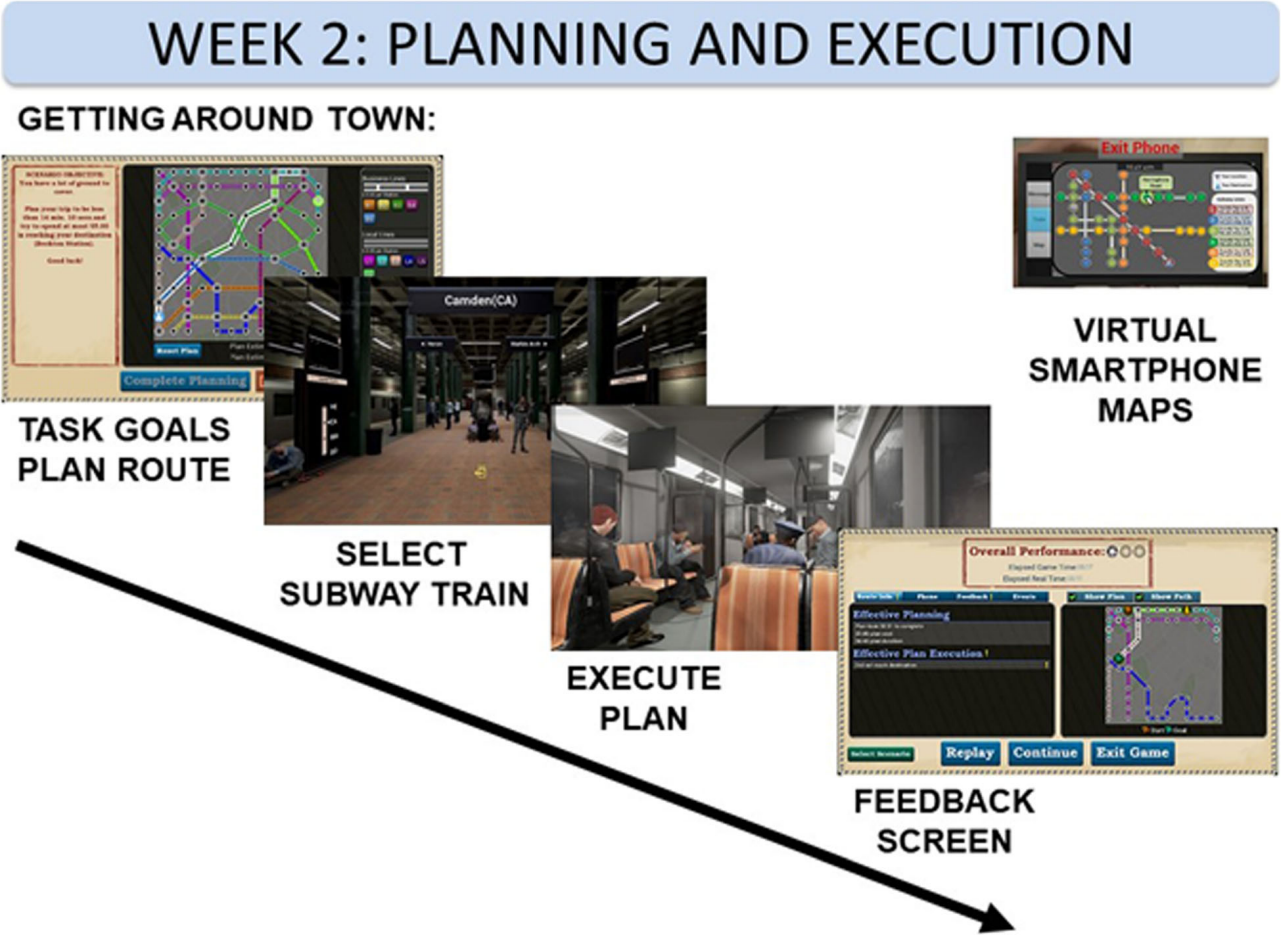
Planning and execution involves determining a route around an unfamiliar city by balancing time and money. Next, participants navigate the route in a virtual transit system by embarking on the correct trains and getting off at stops fitting their plan. Participants are scored on their speed and accuracy, and can view the map on the virtual phone. VR virtual reality

The second week focuses on two constructs: planning and executing a plan. This is operationalized by asking participants to generate a successful sequence and to follow that sequence. Participants are asked to accomplish the functional goal of planning a trip on an unfamiliar subway transit system and then carry out that plan by navigating in a virtual subway car. The planning phase occurs on an interactive transit map in which the participant selects a route that balances time and money (see Fig. 4). After planning their route, participants navigate within a subway system, moving to the correct platforms, boarding the correct trains, and exiting at the appropriate stops in order to carry out their plan.

Participant success, which is based on a proficiency algorithm, determines the difficulty level of subsequent trials. The week 2 challenge level increases by requiring participants to accomplish a more complex plan involving multiple subway lines and with a greater number of stops between the start and the goal. Added difficulty is also implemented in the form of greater distraction during plan execution, as added noise plays on advertising screens in the virtual subway cars and on platforms.

Participants can use the smartphone to access the subway map. This device also serves as extra distraction by buzzing to indicate the arrival of task-irrelevant text messages periodically during the simulation.

#### Week 3

**Fig. 5:**
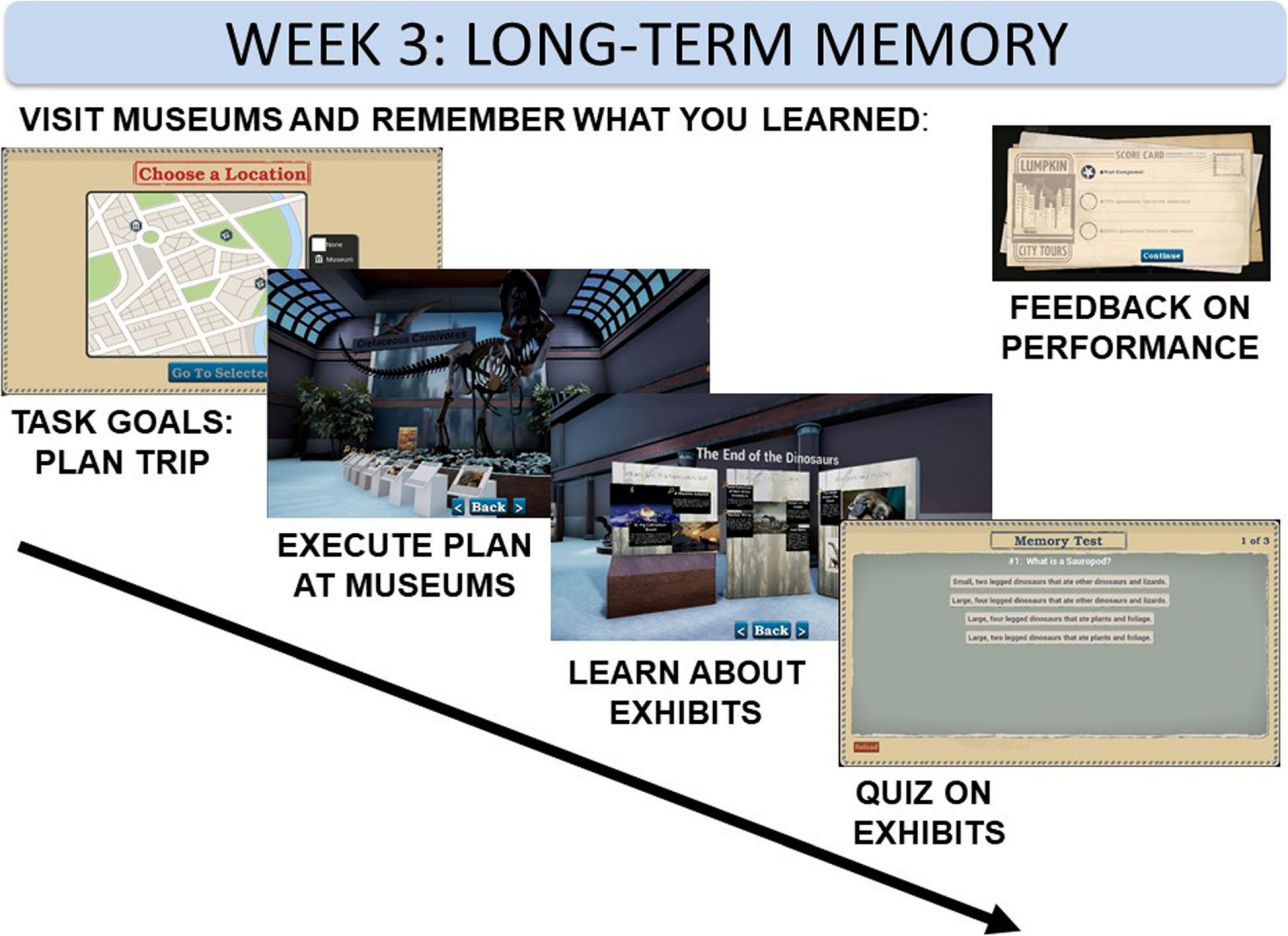
Long-term memory is tested in a series of virtual museums. Participants receive a request that they learn in selected environments. Participants then visit these museums and learn facts at kiosk displays. This module is scored based on a quiz covering what was learned. VR virtual reality

The third week is aimed at improving the construct, long-term memory. This is operationalized through learning new facts. Week 3 taxes the functional goal of effective encoding of new information. Participants are asked to visit a series of virtual museums to view and learn about specified topics. They freely navigate different attractions within multiple museums. After completing their museum visit, they complete a quiz probing their memory for the specific items (Fig. 5).

Difficulty is increased by requiring participants to visit multiple exhibits and multiple museums. Both of these factors increase the overall amount of information that the participants must encode and remember for subsequent memory tests.

As in the prior weeks, the in-game smartphone can be accessed. It can negatively affect performance by buzzing to indicate the arrival of irrelevant text messages to the participants.

#### Week 4

In the fourth week, participants are asked to take on all three scenarios in sequence, as they would in a real-life travel situation (prospective working memory, planning/execution, and long-term memory). Week 4 serves two purposes. First, it demands greater cognitive flexibility upon participants. Second, it emulates an extended task sequence that one could encounter in daily life (pack for a trip, navigate to a location, and visit a destination). This final week may serve as an important indicator of overall success and performance in week 4 may be most predictive of gains on outcome measures.

Participants will be excluded from further participation if they fail to meet the assigned time on the simulations for any given week. We will afford participants an opportunity to pick up on simulation play the following week, but if they do not meet the 5-h criterion we will move to exclude them. If participants are unable to carry out the activities in any of the weeks of the intervention (or are unwilling to), we will discontinue their enrollment. We will make efforts to retain all enrolled participants by reaching out (at least two times per week during the intervention) to ensure that they understand the intervention and are able to complete it. We will also reach out via email and texting if participants are comfortable with those methods.

### Neuroimaging

Each participant is transported a short distance by the experimenter to a nearby building where the MRI scanner is housed. After completing the MRI safety screening form, participants view the experimental task preinstructions under the guidance of the experimenter. Participants are then positioned in the scanner, and the experimenter and a technician run the experiment and scanning protocols, respectively. The protocol for this neuroimaging acquisition is derived from our previous research investigating TBI [29, 30].

#### Functional MRI design

The fMRI Face/Scene Selection Task [31] is performed. The experiment consists of six blocks divided into three runs. All problems follow a similar format: an encoding period showing four images for 4 s total, followed by a 4-s delay and a 2-s probe image. Two are attention blocks, where the participant is instructed to attend to the faces or the scenes with the goal of judging whether the probe image matches and responds with a match/no match button press. One block is a categorization task, where the participant simply presses the right button for a scene and the left button for a face.

Prior to this experimental task, an independent localizer task is used to isolate face and scene selective areas of the brain [31]. The functional localizer includes seven 16-s blocks of grayscale images of faces and scenes.

Lastly, two runs of 7 min (14 min in total) of echo-planar imaging are acquired with participants in a resting state, for which they are instructed to lie still and remain awake in the scanner.

#### Functional MRI data acquisition

Imaging is performed on a 3-T Scanner (Siemens MAGNETOM Prisma). Functional images are acquired with an echo-planar image sequence sensitive to blood oxygen level-dependent (BOLD) contrast (TE 30 ms, TR 2 s, α flip angle 70°). The whole brain is scanned using an 80 × 80 matrix with approximately 37 transverse slices (4-mm thickness with a 0-mm inter-slice gap yielding a voxel size of 3 mm × 3 mm × 4 mm). Three runs of the selective attention task and one run of the localizer task are acquired. Structural images are acquired and these include a magnetization-prepared, rapid-access, gradient-echo image sequence (voxel size 1 mm × 1 mm × 1 mm) and a fluid-attenuated inversion recovery image sequence (voxel size 1 mm × 1 mm × 1 mm).

#### Diffusion tensor imaging design—image acquisition

DTI images are obtained using a single-shot, spin-echo, echo-planar imaging sequence with field of view (FOV) = 224 mm, slice thickness/gap = 2/0 mm, 75 slices, repetition time = 7500 ms, echo time = 65.7 ms, flip angle = 90°, number of excitations (NEX) = 1, a posterior-to-anterior phase encoding direction, and a matrix of 112 × 112. The diffusion-sensitizing gradients are applied at a *b* value of 1000 s/mm^2^ per axis with 40 noncolinear directions and one *b*_0_ image. The acquisition time is 7 min. The voxel size is 2 × 2 × 2. Another *b*_0_ image is also acquired with the opposite phase encoding direction to correct for susceptibility artifacts in the DTI images.

### Assignments to counterbalanced testing–imaging components

New participants are assigned to counterbalanced testing and imaging components by fixed rotation, except when assignments are unbalanced by discontinued participants. In such cases, any underrepresented counterbalance category is filled first.

### Testing and imaging

Each of the testing and neuroimaging sessions occurring in the pretraining phase and the post-training phase is administered by experimenters trained and experienced in their respective procedures.

### Random assignment to groups

To maintain an even balance throughout the study’s duration, participant randomization is completed in runs of 10 individuals using a computerized random number generator to establish a random order of group assignment. Enrolled participants are therefore randomly assigned to either the ESA or the EIA groups prior to their pretesting appointment. Random group assignment is implemented via study personnel who are not involved in participant recruitment, communication, scheduling, or the administration of pretesting and post-training assessments. Group assignment is blinded to all but two of the study personnel via server-side folder-level permissions. Group assignment and participant identification numbers (i.e., randomly generated four-digit numbers that de-identify participants for confidentiality) are only present together during group assignment and within our inhouse database, both of which are blinded to all other study personnel via server-side folder-level permissions.

### Procedures for double blinding

Each experimenter involved in collecting the pretesting, post-testing, and neuroimaging measures is kept blind to the treatment group of each participant. Further, the participants are not informed about the particular content of their treatment group in order to maximize the likelihood that all participants will participate as actively as possible within their particular assigned intervention. Unblinding would only be permitted if a participant’s safety was determined to be at risk.

### Data and statistical analysis plan

#### Power analysis

We performed a sample size analysis to determine the appropriate number of participants to enroll in order to assure that we would achieve adequate statistical power. This was conducted based on data from the primary outcome measure, the VMET (task failures and strategy use), indicating that for an α level of 0.05, an anticipated effect of 0.5 (medium), and a power of 0.8, we need a total of 102 participants (50 in each rehabilitation group) for a one-tailed (ESA > EIA) directional hypothesis. Based on these analyses, we plan to enroll 100 participants. These individuals will be randomized into the two intervention arms.

#### Data management

Cognitive and affective neuropsychological assessment data are scored initially by the study personnel who administered the assessments. The database manager double-checks these data and enters them into an electronic inhouse database. The database automatically crosschecks entered data with its expected permissible values (e.g., numerical entries must be numerical), thus limiting data-entry mistakes. Paper assessments are stored in locked filing cabinets, while digitized and electronically collected data are stored on a password-protected server managed by university technicians. All identifying participant information is password-protected on the university server and no identifying information is stored within the inhouse database. Confidential participant information is never shared with individuals or entities outside the trained study personnel. Audits may be conducted at random by the sponsoring IRBs.

Clinical trial data will also be entered into the Federal Interagency Traumatic Brain Injury Research (FITBIR; https://fitbir.nih.gov) Informatics System in compliance with US Department of Defense funding guidelines. FITBIR is a data-sharing platform designed to facilitate collaborations among the TBI research community and improve research efforts across the country. Identifying participant information is not stored within FITBIR.

#### Statistics for primary outcome measures

Using the Virtual Multiple Errands Test (VMET), we will evaluate intervention efficacy by comparing the performance of the ESA group to that of the EIA group on performance-based measures of executive function ability related to daily life. This first test will evaluate near-transfer of intervention skills to the VMET, a test of executive functions evaluated in a computerized format. The VMET requires working memory, planning, coordinating, and prioritizing, and thus serves as a global, performance-based measure of executive function. We will evaluate the change on the total number of errors from preintervention baseline using a 2 (group) × 2 (testing session) repeated-measures analysis of variance (ANOVA) followed by Bonferroni-correction post-hoc tests to evaluate the significance of simple mean comparisons. We predict a significant time-by-group interaction in which the ESA group will perform more accurately relative to the EIA group at the postintervention test, but not at the preintervention test. In the event that this measure proves insensitive, we will compute and analyze subscores based on strategy errors, completion errors, and rule-violation errors applying an additional Bonferroni multiple comparison correction for the use of three submeasures.

#### Secondary outcome measures

In addition to evaluation of near and far transfer of executive function training skills, we will evaluate a series of subcomponents predicted to be improved by the intervention.

#### Performance on neuropsychological tests

The neuropsychological tests will be evaluated according to the procedures outlined for the use of the ANAM and the relevant scoring procedures for each additional test in the planned battery. These scores will be organized by primary domain and analyzed using 2 (group) × 2 (testing session) ANOVAs. The domains of interest for the neuropsychological battery are as follows: working memory, goal maintenance, verbal fluency, and general cognition. These same groupings will also be applied to the ANAM data, with the exception of verbal fluency as this domain is not examined in our ANAM battery.

#### Multiple correspondence analysis

Multiple correspondence analysis (MCA) is a multivariate statistical method that can analyze a large amount of data from multiple variables and reveal the pattern of relationships and the structure of the data collected from all participants. Projection plot points on each of two graphs represent the observations (e.g., participants) and the variables (e.g., assessments). Projections are mapped onto principal components that account for variance in the data. Projections close together represent similarities according to those components, and distance represents differences. Participants will be grouped in this analysis according to the type of head injury they sustained to determine any patterns in performance deficits (and improvements) for the measures of memory, attention, task switching, and other assessments of executive functioning. We will also include MRI-based data and demographic variables in this MCA, which will enable us to better understand the population and the degree of relatedness among brain, cognitive, life function, and demographic data elements.

#### Analysis of fame performance data acquired during the intervention

Data from the ESA and EIA weekly modules will be analyzed by the total number of task errors and by completion times for each task in order to determine the degree of individual improvement on tasks from day 1 to day 5 during each of the 4 weeks. For the long-term memory module we will also analyze total recall scores across the week. We will apply this same analysis to the overall recall scores obtained in week 4 during the composite game-based simulation. Data will be regularly analyzed by the research team. We do not anticipate needing a data monitoring board, as internal procedures should minimize data fidelity loss. Data will be uploaded to the FITBIR website at regular annual intervals, leading us to attend to the data regularly within the research team. Adverse events will be immediately reported to the IRB agencies sponsoring this trial.

#### Additional neuroimaging methods

##### Functional MRI data acquisition and analysis

Functional images will be acquired on a 3-T Siemens MAGETOM Prisma with an echo-planar image sequence sensitive to BOLD contrast (TE 30 ms, TR 2 s, α flip angle 70°). The volume covers the whole brain with a 64 × 64 matrix and 37 transverse slices (4-mm thickness with a 0-mm inter-slice gap; voxel size 3 mm × 3 mm × 4 mm). The structural scans include a magnetization-prepared, rapid-access, gradient-echo image sequence with 211 sagittal slices and a fluid-attenuated inversion recovery image sequence with 179 slices.

##### Resting-state functional connectivity

Images are preprocessed using AFNI. Standard preprocessing methods include despiking, slice time correction, motion correction, spatial normalization (MNI template), temporal normalization, linear regression, and bandpass filtering (0.009 < *f* < 0.08 Hz). In the linear regression, the rigid head motion profile, the signals averaged over the lateral ventricles, deep cerebral matter, and whole brain, and the first temporal derivatives of the aforementioned parameters are regressed out. After the bandpass filtering, motion “scrubbing” is performed with a frame-to-frame head movement rate of 0.5 mm and a standardized DVARS of 1.8 to prevent potential motion artifacts. The motion “scrubbed” images in the gray matter are then spatially smoothed at 6-mm full-width at half-maximum.

##### Seed-based connectivity analysis

The spatial correlation maps of fMRI images between a 10-mm diameter seed region and the rest of the brain are obtained and then converted by Fisher’s *Z*-transform to enhance the normality. To compare groups on the correlation maps, random-effects analyses are performed on group maps.

##### Brain network analysis

The brain network analysis is performed using the brain connectivity toolbox [32]. Nodes are defined using 264 functional areas, and then edges are defined as the correlation coefficients of time courses between node pairs. Correlation coefficients are thresholded by the strongest proportions of correlations (10 to 2%). Small-worldness, global and local efficiency, centrality, and modularity are evaluated.

##### Diffusion tensor imaging: image acquisition

A 1-shot, spin-echo, echo planar imaging (EPI) sequence is used, with field of view (FOV) = 224 mm, slice thickness/gap = 2/0 mm, approximately 75 slices, repetition time = 7500 ms, echo time = 65.7 ms, flip angle = 90°, NEX = 1, a posterior-to-anterior phase encoding direction, and a matrix of 112 × 112. The voxel size is 2 mm × 2 mm × 2 mm. Images are skull stripped, eddy current corrected, and susceptibility artifacts corrected. Fractional anisotropy (FA), mean diffusivity (MD), and DTI color maps are calculated. FA and MD maps are normalized to Montreal Neurological Institute (MNI) space using a 12-parameter linear registration and subsequent nonlinear registration.

##### Diffusion tensor imaging voxel-based analysis

Normalized FA maps for normal controls are averaged and smoothed with a 4-mm full-width at half-maximum (FWHM) kernel to create a spatially normalized FA template. Subsequently, all FA maps (both intervention groups) are normalized and smoothed with an 8-mm kernel. Voxel-wise statistics are performed on these images using FSL. A two-tailed two-sample *t* test is performed between groups, restricting the analysis to WM voxels using a normalized mask of WM derived from FSL’s MNI Avg152, T1 2 × 2 × 2. WM lesion loads (below normal FA) are calculated using *t* tests between a previously collected control FA map of each individual’s FA maps. The result of each *t* test is a map of voxels with below-normal FA, and these identified voxels will be summed for each participant.

The study IRBs may audit the conduct of this study at any time, although specific scheduled audits are not planned.

## Discussion

This trial investigates training to improve cognition in individuals with mild and moderate chronic TBI. We are evaluating two treatment methods, ESA (active) and EIA (control). The efficacy of these treatments will be evaluated using experimental measures of daily life functioning and neuropsychological measures emphasizing cognitive abilities and neuroimaging measures, including fMRI, DTI, and rsfMRI. The groups are measured prior to training, and immediately after post training. An estimated 59% of military personnel have sustained closed-head injuries. Although many show a rapid and full recovery, as many as 53% experience symptoms long after injury. Many TBI interventions do not jointly address both cognitive and functional impairments. The proposed effort will evaluate the efficacy of VR-based intervention tools for meeting these needs. Telemedicine intervention tools are essential for addressing the underserved military chronic TBI population. Efficacy could lead to large-scale delivery on a national scale.

### Trial status

At the time of the submission of this manuscript (January 29, 2019; Version 2 of protocol), enrollment was ongoing. Enrollment began on July 1, 2018 and is anticipated to carry through June, 2020.

## Data Availability

The datasets generated and/or analyzed during the current study are available in the Federal Interagency Traumatic Brain Injury Research (FITBIR) repository (https://fitbir.nih.gov/). All privacy procedures inherent in the FITBIR policies will be implemented by the study team. Results from this trial will be made available to the general public via publications in scholarly journals, presentations at professional meetings and conferences, and talks for the general public. All authors of such publications must have participated in the design, implementation, and/or write up of the scholarly work as determined to be appropriate by the Principal Investigator.

## Abbreviations

ANAM: Automated Neuropsychological Assessment Metrics
ANOVA: Analysis of variance
ASSIST: Alcohol, smoking and substance involvement screening test
AUDIT: Alcohol Use Disorders Identification Test
BAI: Beck Anxiety Inventory
BDI: Beck Depression Inventory
BOLD: Blood oxygen level dependent
DAI: Diffuse axonal injury
DTI: Diffusion tensor imaging
EIA: Expedition: Informational Advantage
EPI: Echo planar imaging
ESA: Expedition: Strategic Advantage
FA: Fractional anisotropy
FITBIR: Federal Interagency Traumatic Brain Injury Research
fMRI: Functional magnetic resonance imaging
FOV: Field of view
FWHM: Full-width at half-maximum
GCS: Glasgow Coma Scale
GOS-E: Glasgow Outcome Scale—Extended
IRB: Institutional Review Board
MCA: Multiple correspondence analysis
MD: Mean diffusivity
MNI: Montreal Neurological Institute
MRI: Magnetic resonance imaging
NEX: Number of excitations
OSU TBI: Ohio State University Traumatic Brain Injury Screen
PCL: Post-Traumatic Stress Disorder Checklist
PCL-S: Post-Traumatic Stress Disorder Checklist—Specific
PTSD: Post-traumatic stress disorder
rsfMRI: Resting-state functional magnetic resonance imaging
TBI: Traumatic brain injury
TOPF: Test of Premorbid Functioning
VMET: Virtual Multiple Errands Test
